# Advances in sustainable polyolefins: synthesis, chemical transformation and recycling

**DOI:** 10.1093/nsr/nwaf489

**Published:** 2025-11-07

**Authors:** Yuxing Zhang, Dian Yang, Xu Li, Peng-An Chen, Xiukuan Yao, Zhongbao Jian

**Affiliations:** State Key Laboratory of Polymer Science and Technology, Changchun Institute of Applied Chemistry, Chinese Academy of Sciences, Changchun 130022, China; State Key Laboratory of Polymer Science and Technology, Changchun Institute of Applied Chemistry, Chinese Academy of Sciences, Changchun 130022, China; School of Applied Chemistry and Engineering, University of Science and Technology of China, Hefei 230026, China; State Key Laboratory of Polymer Science and Technology, Changchun Institute of Applied Chemistry, Chinese Academy of Sciences, Changchun 130022, China; School of Applied Chemistry and Engineering, University of Science and Technology of China, Hefei 230026, China; State Key Laboratory of Polymer Science and Technology, Changchun Institute of Applied Chemistry, Chinese Academy of Sciences, Changchun 130022, China; School of Applied Chemistry and Engineering, University of Science and Technology of China, Hefei 230026, China; State Key Laboratory of Polymer Science and Technology, Changchun Institute of Applied Chemistry, Chinese Academy of Sciences, Changchun 130022, China; School of Applied Chemistry and Engineering, University of Science and Technology of China, Hefei 230026, China; State Key Laboratory of Polymer Science and Technology, Changchun Institute of Applied Chemistry, Chinese Academy of Sciences, Changchun 130022, China; School of Applied Chemistry and Engineering, University of Science and Technology of China, Hefei 230026, China

**Keywords:** sustainable chemistry, polyolefin, degradation, closed-loop recycling, recyclable polymer

## Abstract

Polyolefins dominate global plastic production due to their cost-effectiveness and versatile performance, yet their environmental persistence and low recycling pose critical challenges to sustainability. Conventional recycling methods of inert native polyolefins face fundamental limitations, including toxic byproduct generation and energy-intensive processes. This review examines advances in sustainable polyolefins that bear weak bonds in terms of synthesis, chemical transformation and recycling. By incorporating cleavable bonds into the polymer backbone, traditionally persistent polyolefins retain essential mechanical properties while enabling designed deconstruction under mild conditions. We critically unify advances across three aspects: synthetic approaches for weak-bond integration; polyolefin main-chain postmodification and transformation; chemical recycling of polyolefin. Recent breakthroughs demonstrate viable routes to sustainable polyolefins. Challenges in structure–property–degradability balancing are analysed, with future directions emphasizing highly controlled degradation, backbone structure optimization and precise control of the polymer-chain structure. This paradigm shift toward degradable-by-design polyolefins offers a roadmap to decouple plastic production from fossil dependence while addressing global plastic recycling.

## Teaser

This review summarizes strategies for incorporating cleavable bonds into polyolefins, enabling their degradation, transformation and chemical recycling under mild conditions to address plastic sustainability.

## INTRODUCTION

Polyolefins have emerged as the most prevalent class of plastics in contemporary industrial applications, owing to their cost-effective production and remarkable versatility across diverse sectors, including packaging, construction and transportation. Global plastics production currently exceeds 400 million metric tons annually, with polyolefins accounting for nearly half of this output [1]. Ethylene-based polymers including high-density polyethylene (HDPE), low-density polyethylene (LDPE) and linear low-density polyethylene (LLDPE) constitute ∼60% of polyolefin production. Market projections indicate a steady annual growth rate of 4%–6% for polyolefins worldwide, sustaining this sector as a vibrant domain for scientific research and technological innovation.

Despite their industrial dominance, polyolefins pose escalating environmental challenges. In 2019, only 9% of the global plastic waste entered recycling streams. Approximately 50% accumulated in landfills, while another 20% was mismanaged, escaping formal waste systems and threatening ecosystems [2]. Continued reliance on virgin feedstock exacerbates petroleum dependence and environmental pollution. To reconcile consumption demands with planetary boundaries, a global transition from linear to circular economic models is imperative.

The low recycling rate stems from both systemic management flaws and technological limitations [3]. Conventional recycling paradigms fail to resolve these sustainability crises. While energy recovery via plastic incineration yields 20–47 MJ/kg of thermal output (comparable to petroleum) [4], incinerating mixed waste generates toxic byproducts (e.g. dioxins), creating secondary environmental burdens. Mechanical recycling—entailing waste collection and reprocessing—faces intrinsic constraints: economic viability, degradation of mechanical properties and inconsistent product quality. Real-world plastic-waste heterogeneity further complicates recycling; e.g. multi-material packaging (e.g. snack bags, beverage cartons) combines plastics, metals and paper in inseparable layers to achieve enhanced functionality [5,6]. Existing systems cannot economically separate these complexes, resulting in recycled materials with compromised properties that are relegated to low-value applications. Even with compatibilizers, output quality often remains subpar and the sorting infrastructure remains inadequate for hybrid material streams.

Chemical recycling, which depolymerizes polymers into monomers or high-value chemicals, offers a promising alternative. For polyolefins such as HDPE, depolymerization is thermodynamically possible at very high temperatures, but such temperatures exceed the degradation onset of the polymers. Consequently, pyrolysis, with low selectivity, occurs before depolymerization can take place and yields <25% of reusable monomers alongside complex hydrocarbon mixtures that require energy-intensive separation [7]. Recently, catalytic cracking mediated by inorganic acids has been reported to enable the degradation of inert polyethylene (PE) under mild conditions (∼200°C), yielding a mixture of saturated and unsaturated hydrocarbon liquids (C1–C7) at a high conversion rate of >90 wt% [8]. Although the selectivity of the products can be partially controlled through the design of the catalyst, the resulting mixture remains highly complicated [9].

Consequently, research focus has shifted toward reinventing PE via molecular engineering. Recent breakthroughs demonstrate that strategically incorporating weak bonds—ester linkage, ketone or unsaturation—into hydrocarbon backbones preserves mechanical properties while enabling designed recyclability. For example, introducing ester groups permits depolymerization at 120°C [7], slashing energy inputs by >75% compared with those for pyrolysis.

**Figure 1.**
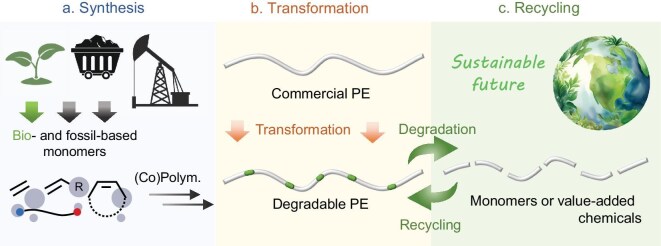
Scope of this review. (a) Synthesis of PE-type materials from fossil- or bio-based monomers. (b) Post-use polymer transformation to produce degradable PE and value-added chemicals. (c) Recycling of PE-type materials for sustainable future.

Driven by these advances, sustainable polyolefin research has gained significant momentum. To address the dual challenges of environmental persistence and recycling inefficiency delineated in this introduction, this review summarizes recent breakthroughs in strategically engineering polyolefin-type polymer-chain architectures through weak-bond incorporation—a transformative paradigm that maintains material performance while enabling energy-efficient degradation. This review critically unifies advances across three aspects (Fig. 1):

- Synthesis: strategic incorporation of weak bonds into polyolefin main chains (carbonyl, esters, double bonds, mechanophores, etc.).
- Transformation: chemical transformation of commercial or degradable polyolefins into recyclable polyolefins or high-value chemicals.
- Recycling: closed-loop chemical recycling of degradable polyolefins.

By systematically examining synthesis, transformation and recycling, this review not only clarifies current scientific understanding, but also charts a course for next-generation breakthroughs.

## SYNTHESIS OF DEGRADABLE POLYOLEFINS

The exceptional stability of the C–C bonds in polyolefins confers outstanding thermal stability and weatherability, but simultaneously hinders their sustainable end-of-life management. A promising strategy to achieve sustainable polyolefins involves synthesizing materials containing main-chain ‘weak bonds’—such as carbonyl groups (keto-group), ester linkages, unsaturated bonds, etc., which are tailored for controlled deconstruction or transformation. These cleavable bonds are strategically incorporated at low densities via copolymerization, preserving the intrinsic properties of PE while endowing it with desired degradability.

### Keto-based degradable polyolefins

**Figure 2.**
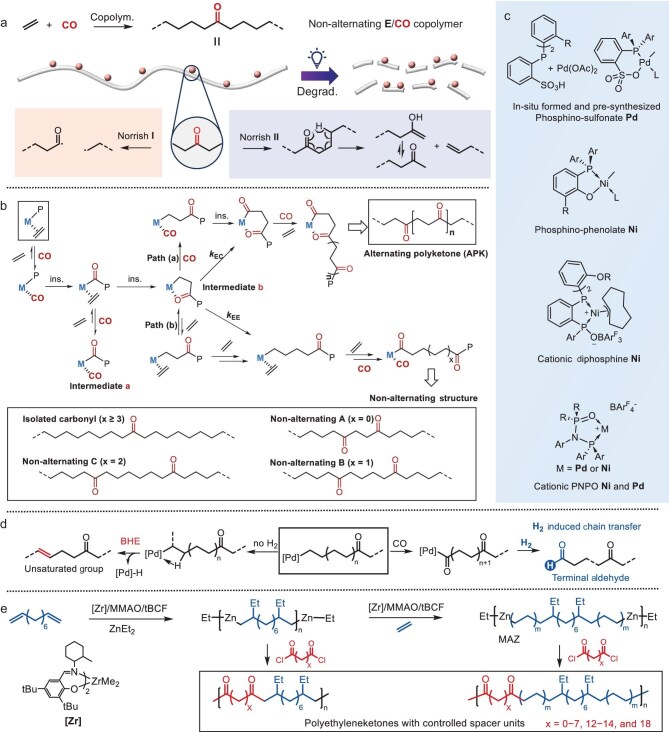
(a) Synthesis and degradation of non-alternating E/CO copolymer. (b) Mechanism of E/CO copolymerization and four types of non-alternating structures in non-alternating E/CO copolymers. (c) Typical catalysts for E/CO non-alternating copolymerization. (d) H_2_-induced chain transfer producing terminal aldehyde (BHE = β-H elimination) [19]. (e) Polyethyleneketones with controlled spacer units [22].

The strategic incorporation of carbonyl groups into polyolefins, typically PE backbones, represents a significant advancement in sustainable PE design, balancing material performance with end-of-life degradability. Ethylene/carbon monoxide (E/CO) copolymerization yields non-alternating copolymers in which isolated ketone groups confer intrinsic photodegradability through Norrish reactions while maintaining excellent PE-like properties (Fig. 2a). Upon light exposure, these carbonyl functionalities undergo either Norrish type I (radical-mediated) or type II cleavage (γ-hydrogen transfer via a six-membered cyclic transition state), with the latter dominating (>90% under ambient conditions) to produce methyl ketones and terminal vinyl groups [8]. This photodegradation mechanism, elucidated through radical-synthesized copolymers, enables material degradation while preserving mechanical integrity during useful life.

Commercial implementation dates to DuPont’s pioneering high-pressure radical copolymerization (1940s), operating at >1000 atm in either tubular or stirred autoclave reactors to produce lightly branched resins containing 0.5–4.0 wt% CO (Table 1, entry 1), using techniques similar to those used to make high-pressure LDPE. These LDPE-analog materials gained prominence in the 1970s for photodegradable applications such as six-pack ring carriers, leveraging their controlled environmental breakdown [9]. Recent innovations by Mecking and co-workers have improved process sustainability, achieving copolymerization at moderate pressures (<350 atm) by using green solvents (dimethyl carbonate/water) with commercial initiators or organocobalt(III) [10,11].

**Table 1.** Four types of E/CO copolymers.

Entry	E/CO copolymer structure	Synthesis^a^	Topologies	Structure	Main structure	*T*_m_ (°C)	Ref.
1	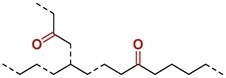	RP	Branched	Non-alternating	PE	52–119	[10,11]
2	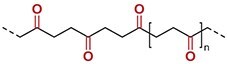	CIP	Linear	Alternating	Polyketone	228–266	[12,13]
3	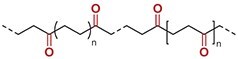	CIP	Linear	Non-alternating	Polyketone	183–246	[14–16]
4	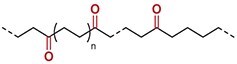	CIP	Linear	Non-alternating	PE	130–136	[17–20]

a RP, radical polymerization; CIP, coordination–insertion polymerization.

While radical copolymerization effectively incorporates carbonyl groups, its inherent branching limits thermal properties (*T*_m_ < 120°C), precluding potential applications such as HDPE. Mecking *et al.* employed an acyclic diene metathesis (ADMET) approach by using docosa-1,21-dien-11-one and undeca-1,10-diene, followed by hydrogenation to yield strictly linear chains with low-density in-chain ketones [21]. These architectures exhibit a high melting point (125–134°C), which significantly surpasses those of both radical-synthesized analogs and linear aliphatic polyesters or polycarbonates.

A transformative breakthrough came in 2021 with neutral phosphino–phenolate Ni(II) catalysts (Fig. 2c), which achieved the long-sought non-alternating copolymerization under low CO concentrations (≤0.6 mol%) [17,28]. These systems produced high-molecular-weight polymers (≤215 kDa) containing exclusively isolated carbonyls and a non-alternating A structure, as shown in Fig. 2b (0.3–3.1 mol% CO), with exceptional HDPE-like characteristics: crystallinity of ≤71%, *T*_m_ ≥ 133°C and retained photodegradability (Table 1, entry 4). The stark contrast with salicylaldiminato Ni(II) catalysts—which exclusively yield alternating sequences—highlights the critical role of the ligand design in controlling insertion kinetics. Water-soluble catalyst variants further enabled the aqueous-phase synthesis of particulate photodegradable PE-type materials with ≥70% crystallinity and *T*_m_ ≥ 132°C [29].

Fundamental kinetic barriers to non-alternating copolymerization were further elucidated through cationic diphosphine Ni(II) catalysts (Fig. 2c) [30]. While initially appearing to produce low-CO-incorporation copolymers, detailed characterization revealed that these materials were actually blends of alternating polyketones and minimally functionalized PE—a consequence of CO’s extreme binding affinity to cationic Ni(II) centers. This work established a critical design principle: when the kinetic preference for alternating sequences (*k*_EC_/*k*_EE_) is high (Fig. 2a), the operational window for non-alternating products becomes impractically narrow due to uncontrollable composition drift. Comparisons highlighted stark contrasts: phosphine–sulfonate Pd(II) systems (*k*_EC_/*k*_EE_ ∼ 10^2^) permit viable non-alternating synthesis, whereas cationic diphosphine Ni(II) catalysts (*k*_EC_/*k*_EE_ ∼ 10^4^–10^5^) inherently favor alternating polyketone production [30]. Recently, Mecking and Caporaso achieved a breakthrough by using sterically demanding phosphine–sulfonate Pd(II) catalysts with continuous CO feeding, demonstrating the scalable production of homogeneous E/CO non-alternating copolymers [20].

Complementary advances emerged from cationic diphosphazane monoxide (PNPO) catalysts (Fig. 2c), which exhibit remarkable productivity in alternating copolymerization [13–15,31–33]. Nickel-based PNPO variants achieved limited non-alternation (≥40% alternating carbonyl) even under forcing conditions (130°C, 0.2 mol% CO), while their palladium counterparts unexpectedly produced fully non-alternating architectures [34]. Beyond E/CO copolymerization, this catalyst platform enables terpolymerization with α-olefins to synthesize high-CO polyolefins (CO incorporation ≥42 mol%) [12,32].

Terpolymerization strategies significantly broaden the functional scope of carbonyl-containing PE. Phosphino–phenolate Ni(II) systems (Fig. 2c) exhibited increased chain transfer during E/CO/methyl acrylate terpolymerization, reducing the molecular weights versus those in E/CO copolymers [35]. Jian *et al.* reported the phosphine–sulfonate Pd(II)-catalysed terpolymerization of ethylene/CO with diverse polar monomers—including acrylates, acrylic acid and vinyl ethers—yielding high-molecular-weight linear polymers with >99% isolated ketones alongside ester, ether and cyano functionalities without the chain transfer induced by polar monomers [36]. Norbornene incorporation conversely enhanced ductility while progressively lowering the melting points relative to those of binary E/CO analogs [37]. Beyond comonomers, Jian *et al.* utilized a syngas component in terpolymerization (ethylene/CO/H_2_), which exploits H_2_ as a chain-transfer agent, regulating the molecular weight (43–195 kDa) while replacing typical olefinic end groups with unique aldehyde termini (Fig. 2d) [19].

Coordination copolymerization offers a more industrially viable route to linear architectures by using economical ethylene/CO feedstocks. However, CO’s strong binding affinity creates dormant intermediates (Fig. 2b, Intermediate a), favoring alternating insertion pathways (Fig. 2b, Path a) that produce high-melting-point polyketones (228°C–266°C, Table 1, entry 2) rather than desirable PE analogs (Fig. 2b, Path b; Table 1, entry 4) [23]. Overcoming this kinetic preference requires catalysts with attenuated CO affinity. In 2002, Drent and Pugh’s pioneering phosphine–sulfonate Pd(II) system (Fig. 2c) achieved the first non-perfectly alternating copolymers by enabling extra ethylene insertion at elevated temperatures (Table 1, entry 3) [16]. Subsequent work revealed that both temperature and CO/C_2_H_4_ feed ratios critically modulate the sequence distributions [24–27].

Innovative carbonyl-sourcing strategies further diversify the copolymerization. Nozaki’s group reported a metal carbonyl approach enabling gradual CO release for isolated-ketone incorporation (0.14–3.9 mol%) via neutral phosphino–phenolate Ni(II) [38] and phosphine–sulfonate Pd(II) [18] catalysts. Cyclopropenone serves a dual role: ring-opening copolymerization installs α,β-unsaturated ketones while simultaneously releasing CO for backbone ketone incorporation [39]. Recently, the coordinative chain-transfer polymerization (CCTP) of α,ω-dienes with ZnEt_2_/ethylene has produced telechelic Zn-PEs [40] that undergo diacid chloride functionalization to create perfectly spaced ketones with programmable spacers (Fig. 2e) [22]. Photodegradation studies of these ‘polyethyleneketones’ have revealed spacer-length-dependent kinetics: C6–C18 spacers enable efficient cleavage while C3–C5 spacers impede degradation—highlighting the critical role of the polymer-chain sequence in Norrish reactivity.

Sustainable monomer sourcing reaches its zenith in carbon dioxide (CO_2_ as CO source) utilization strategies. Miller, Liu and Wang’s electrochemical/organometallic cascade coupled CO_2_ electroreduction (producing CO) with E/CO copolymerization to produce polyketones and non-alternating E/CO copolymers [41]. Tang and Zhou integrated photocatalytic CO_2_ reduction and Pd(II)-catalysed E/CO copolymerization within a single reactor [42]. Agapie and Peters achieved fully *in situ* alternating E/CO copolymerization in which both monomers derived electrochemically from CO_2_ and H_2_O—an idealized route to carbon-negative polymers despite current efficiency limitations [43]. This method for producing degradable polyolefins by using carbon dioxide and water as feedstocks highlights the principles of green chemistry. Although its current low reaction efficiency limits practical application, it explores a sustainable pathway for the future development of polymers. Further research should focus on improving reaction efficiency and cost control, which are crucial for the practical viability of this approach.

The majority of previous studies on keto-based polyolefins have primarily focused on the effective synthesis of non-alternating copolymers. Very recently, Nozaki *et al.* investigated the photodegradation behavior of two carbonyl-containing polyolefins: a non-alternating E/CO copolymer with in-chain carbonyl groups and an ethylene/methyl vinyl ketone (E/MVK) copolymer featuring pendant carbonyl functionalities. Under 275 nm of ultraviolet (UV) irradiation, the E/MVK copolymer (1.57 mol% MVK) degraded more rapidly than its E/CO counterpart (1.70 mol% CO), with the former’s weight-average molecular weight (*M*_w_) decreasing from 14.2 to 2.5 kDa over 28 days, compared with the latter’s more gradual reduction from 9.8 to 4.0 kDa. During the degradation of E/MVK, several radical-mediated side reactions induced by Norrish type I cleavage were observed, including backbone hydroxylation, carbonyl formation and even cross-linking (at a high MVK concentration of 4.37 mol%) [47]. Moreover, when it was blended with HDPE, this free-radical reaction can contribute to the degradation of HDPE.

Given the long-time degradability, these keto-based polyolefins may be utilized for the production of disposable plastic packaging. The sustainability of post-degradation products remains a weak point in this area. Moving forward, efforts should focus on enhancing the controllability of photodegradation to tailor the structure of degradation products and explore their potential for reuse. Besides, given that the molecular weights of these photodegradation products were typically in the thousands, systematic assessment of their environmental fate will be critical to ensuring the viability of these photodegradable PEs as truly environmentally friendly and sustainable alternatives. Microbial degradation, particularly adept at decomposing into low-molecular-weight oligomers, may offer a viable solution to this issue [48,49].

### Ester-based degradable polyolefins

Despite the functionality of carbonyl-functionalized polymers for UV-light-induced degradation, their reaction time is notably protracted, often spanning several days or even several months [17,18]. Furthermore, the degradation products of these polymers do not possess the capability to be repolymerized into their original forms. Conversely, the incorporation of reversible ester bonds facilitates straightforward chain scission and permits repolymerization (discussion in the ‘Chemical recycling of polyolefins’ section) [7,44]. In addition to promoting recycling, the polarity of the ester group also improves the surface properties.

#### Degradable polyolefins from biomass resources

**Figure 3.**
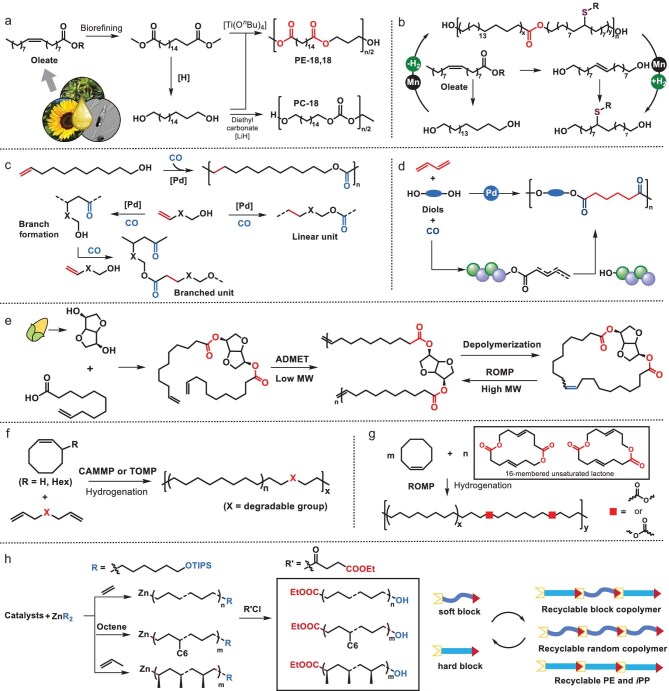
Synthetic strategies for in-chain ester incorporation into PE. (a) Transesterification polycondensation (AA + BB-type) of bio-derived C_18_ diols/diesters yielding long-chain aliphatic polyester (PE-18,18) and polycarbonate (PC-18) [7,45,47,50]. (b) Acceptorless dehydrogenative polymerization (AA + AA-type) of bio-derived diols using Mn-based pincer catalyst [55]. (c) Competing pathways in alkoxycarbonylation: linear polyester formation versus carbonylative branching [60]. (d) Catalytic isomerizing dual hydroesterification of 1,3-butadiene/diols producing poly(adipate) esters [61]. (e) Construction of in-chain-ester polyolefin-like thermoplastic elastomer via metathesis homopolymerization of fully bio-derived monomer [62]. (f, g) Ring-opening metathesis polymerization producing degradable polyolefins [68–70]. (h) CCTP with functional ZnR_2_ agents producing AB-type telechelic polyolefins for subsequent AB + AB transesterification polycondensation to recyclable PE, isotactic polypropylene (*i*PP) and olefin block copolymers (OBC) [71,72].

The incorporation of ester linkages into polyolefins, typically PE backbones, primarily centers on the stepwise polycondensation of telechelic macromonomers—sourced through biomass conversion. A paradigm example employs bio-renewable C_18_ building blocks: AA-type 1,18-octadecanedioate and BB-type octadecane-1,18-diol derived from plant oil biorefining, enabling the synthesis of aliphatic polyester-18,18 (PE-18,18) [45,46] and polycarbonate-18 (PC-18) [47] via AA + BB polycondensation (Fig. 3a) [48–51]. Mecking *et al.* demonstrated that these long-chain aliphatic polymers achieve substantial molecular weights (PC-18: *M*_w_ ∼ 300 kDa; PE-18,18: *M*_w_ ∼ 80 kDa) [7], exhibiting HDPE-equivalent elastic moduli and ductility. However, their melting points are significantly reduced (PE-18,18: *T*_m_ ∼ 100°C; PC-18: *T*_m_ ∼ 85°C vs. HDPE’s 130°C) due to ester-induced crystalline disruption [52].

Beyond classic polycondensation, dehydrogenative polymerization [53] offers an alternative esterification pathway. Miyake’s group reported ruthenium (Ru) [44,54] and Earth-abundant manganese (Mn) [55] catalysts for the AA + AA-type coupling of telechelic diols, circumventing stoichiometric constraints (Fig. 3b). These diols could be obtained from oleate [55] or ring-opening metathesis polymerization (ROMP) with chain-transfer agents [44,54]. By combining hard/soft diols, this method synthesizes recyclable multiblock PE with tunable mechanical properties (elastomer to plastomer to thermoplastic) and exceptional thermal profiles: high melting transitions (*T*_m_ ≤ 128°C) coupled with low glass transitions (*T*_g_ as low as −60°C).

Alternatively, ester linkages can be constructed via the palladium-catalysed alkoxycarbonylation of alkenes, alcohols and CO [56,57]. This process proceeds through a Pd–(CO)–alkyl (metal acyl) intermediate that reacts with the alcohol group of monomers to form linear aliphatic polyesters (Fig. 3c). Mecking *et al.* demonstrated this by using bio-based undec-10-ene-1-ol [56,57], achieving linear polyesters with *M*_n_ of ≤17.4 kDa and *T*_m_ reaching 75°C with minimal isomerization (Fig. 3c), though constrained molecular weights suggest chain-growth inhibition by side reactions, including alcoholic end-group oxidation, hydroxycarbonylation and acetylation [58]. Tonks *et al.* expanded the monomer scope through undecenol etherification [59], while mechanistic studies revealed ligand-dependent competitive pathways: alkene hydroesterification yields linear ester units, but alternating alkene/CO copolymerization generates keto/branched alcohol groups that form branched esters [60] (Fig. 3c). Recently, Liu, Tang and co-workers reported that Pd(II)-catalysed isomerizing dual hydroesterification [61] (Fig. 3d) enabled the direct conversion of 1,3-butadiene/diols/CO into poly(adipate) esters with exceptional linear selectivity (>20 : 1 linear/branched ratio) through efficient isomerization-controlled repeating unit formation.

Jia, Sha and co-workers developed a fully bio-based diene monomer containing ester linkages, synthesized from isomannide and undecenoic acid [62] (Fig. 3e). While the ADMET homopolymerization of this monomer yielded only low-molecular-weight polymers (*M*_n_ ≤ 23.3 kDa), these oligomers could undergo ring-closing metathesis depolymerization to form cyclic olefins. These cyclic olefins were then successfully repolymerized via ROMP, affording high-molecular-weight polymers (*M*_n_ ≤ 794.2 kDa). The resulting polymers demonstrated excellent thermal stability (*T*_d_ = 375°C–426°C) and exhibited characteristic elastomeric behavior. Notably, this system features dual degradation pathways: hydrolytic cleavage regenerates telechelic diacid and isomannide, while metathesis-based cyclodepolymerization provides an alternative degradation mechanism.

#### Degradable polyolefins from metathesis polymerization

Olefin metathesis copolymerization [63] offers a versatile platform for the incorporation of ester groups into polymer backbones. Using Grubbs-type metathesis catalysts, ester functionalities can be introduced through two primary approaches: (i) ROMP of cyclic olefins with cyclic unsaturated lactones [64–66]; or (ii) ADMET polymerization of non-cyclic dienes with ester-containing dienes [67], followed by subsequent hydrogenation.

In 2022, Chen and colleagues pioneered an innovative approach through cyclic–acyclic monomer metathesis polymerization (CAMMP) [68], which enables the incorporation of various weak bonds, including multiple types of ester linkages, within PE chains. This strategy is particularly advantageous because it utilizes commercially available or readily synthesizable diene comonomers under standard ROMP conditions. However, it should be noted that the incorporation of polar dienes inherently limits the attainable molecular weight and precludes molecular-weight control (Fig. 3f). In 2024, the same group successfully addressed this molecular-weight limitation by developing a tandem olefin metathesis polymerization approach [69]. This method combines the ring-closing metathesis of diene comonomers with the subsequent ROMP of cyclic olefin monomers. Remarkably, this integrated strategy achieves both transformations by using a single metathesis catalyst, yielding closed-loop recyclable polyolefins with outstanding material properties.

Recently, Jian *et al.* advanced this paradigm by using a rationally designed 16-membered unsaturated lactone comonomer [70] (Fig. 3g). Through copolymerization with cyclooctene followed by hydrogenation, HDPE-like materials were produced, preserving the bulk mechanical properties, yet readily degrading into stoichiometrically self-balanced telechelic macromonomers via hydrolysis. Crucially, the macromonomer molecular weight was tunable via the cyclooctene/lactone feed ratio—confirming an even ester distribution and enabling the tailored synthesis of circular PE [70].

#### Degradable polyolefins from coordination polymerization

Complementary to biomass-derived routes, telechelic macromonomers are accessible through CCTP [40]. Tang, Chen, Gao and co-workers pioneered this approach by using the functionalized alkyl zinc reagent Zn[(CH_2_)_6_OTIPS]_2_ (TIPS = triisopropylsilyl) in ethylene/1-octene CCTP [71]. Post-polymerization acylation efficiently converted the zinc termini into ester functionalities, achieving >99% selectivity through a palladium-catalysed end-capping reaction [72]. This process yielded PE-based AB-type telechelics with –OH and –CO_2_Et end groups (Fig. 3h). These building blocks enabled precise AB + AB transesterification polycondensation, producing degradable PE, isotactic polypropylene (*i*PP), olefin block copolymers, random copolymers and multiblock PE/*i*PP architectures. Notably, the inherent 1 : 1 stoichiometry eliminates feed ratio imbalances, yielding recyclable *i*PP with near-conventional thermal properties (*T*_m_ ∼ 146.9°C vs. commercial *i*PP 153.5°C) and mechanical performance [72].

### Unsaturated-group-based degradable polyolefins

**Figure 4.**
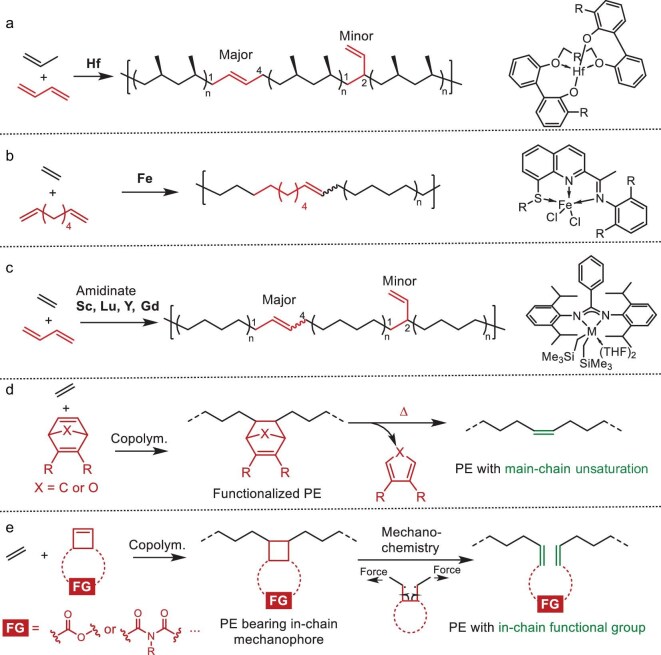
Strategies for in-chain unsaturation and other functionalities incorporation via coordination–insertion copolymerization. (a) Isoselective copolymerization of propylene and butadiene yielding unsaturated *i*PP [73]. (b) Ethylene copolymerization with non-conjugated α,ω-dienes using Fe(II) catalysts [74]. (c) Ethylene/butadiene copolymerization producing unsaturated PE [75]. (d) Retro–Diels–Alder reaction produces main-chain unsaturation [76,77]. (e) PE materials bearing in-chain mechanophores enables in-chain functionalization [78–80].

The reactivity of in-chain double bonds enables controlled polyolefin degradation through metathesis pathways. The incorporation of an in-chain unsaturated group could be achieved via the copolymerization of ethylene or propylene with dienes (conjugated [73,75,81–84] or non-conjugated dienes [74]) or through the dehydrogenation of PE [82,85] (discussed in the ‘Degradable polyolefins from chemical transformation of PE’ section). A significant advancement came in 2022 with Coates, Laphin and co-workers’ development of bridged biphenylphenol hafnium catalysts for isoselective propylene/butadiene copolymerization (Fig. 4a) [73]. This system achieved ∼80% regioselective 1,4-insertion while maintaining moderate molecular weights. The incorporation of 0.2–0.34 mol% of butadiene reduced the *i*PP’s melting temperature to 90°C–110°C, yielding materials with LLDPE-like thermal and mechanical properties. Crucially, these unsaturated copolymers underwent metathesis depolymerization with functional mono-olefins (2-hydroxyethyl acrylate) to telechelic macromonomers, which, upon hydrogenation and repolymerization, generated ester-linked PPs mirroring the original properties—demonstrating closed-loop recyclability.

Parallel advances by Huang, Guan, Liu and co-workers demonstrated thio–imino–quinoline Fe(II) complexes enabling PE in-chain unsaturation through ethylene/non-conjugated α,ω-diene copolymerization (Fig. 4b) [74]. This proceeds via a distinctive mechanism: sequential 2,1-insertion of the diene’s first vinyl group into the Fe–C bond, β-hydride elimination and 1,2-insertion of the second vinyl group into the Fe–H bond. While mechanistically elegant, the approach yielded relatively low-molecular-weight copolymers (<5.5 kDa). Agapie’s group advanced the field in 2023 by using titanium bisphenoxide bisthiolate catalysts for ethylene/butadiene copolymerization, in which high 1,4-selectivity enabled subsequent metathesis-driven ethenolysis to α,ω-dienes (C10–C20 range) [81]. In parallel, Liu and Cui achieved controlled ethylene/butadiene copolymerization via an amidinate rare-earth metal, such as gadolinium (Gd) complexes (Fig. 4c) [75], attaining 86% 1,4-insertion with 2.4 mol% butadiene incorporation. The resulting copolymers retained HDPE-equivalent physical, mechanical and processing properties while crucially preserving commercial-grade antioxidative stability. In-chain unsaturation permitted metathesis degradation to narrowly dispersed α,ω-telechelic oligomers by using functional mono-olefins, which subsequently served as macroinitiators for atom transfer radical polymerization (ATRP) or immortal ring-opening polymerization (ROP)—demonstrating efficient pathways to triblock copolymer compatibilizers and validated upcycling potential.

The reversible nature of the Diels–Alder (DA) reaction has been widely exploited in dynamic cross-linking materials [86]. Typically, substituted pentadiene or furan derivatives react with dienophiles to form norbornene derivatives. These adducts can undergo retro-DA reaction upon heating, regenerating the double bond in the polymer backbone while releasing the pentadiene or furan derivative byproducts. Coates *et al.* reported the synthesis of norbornene-functionalized HDPE via the copolymerization of ethylene with dimethyl 7-oxabicyclo[2.2.1]hepta-2,5-diene-3,5-dicarboxylate (Fig. 4d, *X* = O) [76], yielding copolymers with comonomer incorporation of 0.79–2.24 mol% and *M*_n_ ∼ 29 kDa. These copolymers could undergo dehydrogenation to form in-chain *o*-arylene structures [87]. Alternatively, performing a post-polymerization retro-DA reaction at 165°C unveiled the hidden double bonds within the polymer backbone, producing unsaturated HDPE with controlled segment lengths (1.2, 1.9 and 3.5 kDa) between the double bonds [76]. This unsaturated PE can undergo further degradation via cross-metathesis reactions (discussed in the ‘Chemical transformation of degradable polyolefins’ section).

However, when the comonomer scope was expanded to norbornene derivatives with a carbon bridge atom instead of oxygen (Fig. 4d, *X* = C), although the lower polarity facilitated higher incorporation, the corresponding copolymers exhibited retro-DA temperatures of >250°C. Under these high-temperature conditions, uncontrolled cross-linking occurred, leading to insoluble products. By employing antioxidants with higher thermal-decomposition temperatures, Jian’s group achieved a complete retro-DA reaction for copolymers featuring the carbon-bridged norbornene derivative (Fig. 4d, *X* = C) at 270°C [77]. This reaction was conducted under a vacuum in a high-boiling-point solvent (b.p. ∼ 40°C) to effectively remove the liberated byproducts to promote the reaction. The resulting polymer contained ≤28.7 mol% of unsaturated bonds, which could be further degraded into long-chain α,ω-dienes and C9/C9+ hydrocarbon products. This chemical upgrading and recycling approach enhances the sustainability of PE-type plastics.

### Mechanophore-based degradable polyolefins

In contrast to the above-mentioned cleavable bonds, which require external chemical reagents or may produce byproducts, mechanophore activation [88] relies solely on mechanical force. Mechanophores embedded within the polymer backbone can be categorized into two types: scissile or non-scissile [89]. Upon force activation (e.g. via ultrasonication or ball milling), scissile mechanophores (such as anthracene dimers and coumarin dimers) cleave the polymer chain into fragments. Conversely, non-scissile mechanophores (e.g. spiropyran) undergo structural changes without chain scission [90], ensuring the stability of the polymer performance. The high activation energy barrier for unsubstituted cyclobutane cycloreversion (58–60 kcal/mol) [90] ensures its thermal stability, making cyclobutane-based mechanophores particularly suitable for balancing the thermostability, mechanical properties and degradability in PE-type materials.

Through the palladium-catalysed coordination–insertion copolymerization of ethylene and cyclobutene derivatives, PE bearing in-chain cyclobutane-fused polar monomers has been synthesized [79,80] (Fig. 4e). Several cyclobutane-fused mechanophores were successfully incorporated (0.35–26 mol%). While the incorporation reduced the copolymer melting point (*T*_m_ = 121°C–105°C, cf. HDPE: *T*_m_ = 135°C), it significantly increased the tensile strength (∼40 MPa, cf. HDPE: ∼15 MPa), indicating copolymer stability during standard melt processing and mechanical deformation [79]. Upon mechanochemical activation (ultrasonication or ball milling), degradable functional units (e.g. imide and ester groups) can be introduced into the main chain. This transformation alters the polymer backbone, thereby enhancing its degradability [91,92]. Critically, combining mechanochemical activation with acid hydrolysis enables the efficient degradation of high-molecular-weight PE materials into telechelic oligomers [78–80]. While the transformation of mechanochemical groups is challenging to control with precision (high selectivity and high proportion of conversion remains difficult), PE materials incorporating in-chain mechanophores offer an attractive and promising combination of desirable material properties and accessible degradability pathways.

### Other functional group-based degradable polyolefins

In addition to the aforementioned reversible bonds, metathesis copolymerization [63] provides a versatile platform for incorporating diverse cleavable functionalities into polymer backbones, including orthoesters [93], carbonates [94], Si–O bonds [95–98], S–S bonds [99,100], acylsilanes [101], acetals [102] and thioacetals [103], etc. Upon hydrogenation, these polymers can achieve polyolefin-like mechanical properties while their degradability is preserved. Johnson *et al.* synthesized a degradable polyolefin with HDPE-like mechanical strength via the ROMP of a cycloolefin monomer containing O–Si–O linkages, followed by hydrogenation [97]. The O–Si–O bonds can then be selectively cleaved under acid catalysis, generating telechelic PE with dihydroxy end groups—without disrupting the carbon–carbon backbone or ester linkages. This selective cleavage enables the efficient recovery of the target polymer from mixed-plastic waste (PP, PE and polyethylene terephthalate (PET)). Moreover, the resulting hydroxy-terminated telechelic PE can be repolymerized with dialkyldichlorosilanes, effectively closing the recycling loop and regenerating the original polymer.

From an engineering perspective, sustainable polyolefins synthesized via the ROMP of cyclic olefins followed by hydrogenation are currently limited to serving as model polymers for studying structure–property relationships, rather than being a viable route for large-scale production, primarily due to the high costs of feedstock and catalysts. In contrast, coordination polymerization using ethylene and bulk monomers such as butadiene or carbon monoxide offers a more cost-effective alternative. This approach is also compatible with the existing industrial polymerization infrastructure, such as slurry-phase reactors. However, the key challenge lies in the post-use degradation and recycling of these materials, particularly their economic feasibility and environmental impact. This aspect has been discussed in the ‘Keto-based degradable polyolefins’ and ‘Unsaturated-group-based degradable polyolefins’ sections, with special emphasis on photodegradable PE. Alternatively, degradable polyolefins with ester or carbonate groups in the backbone can be synthesized via polycondensation, which can be directly scaled up by using existing polyester, such as PET, production lines. The primary consideration for this route is the sourcing of diols and diacids/diesters, making feedstock cost control a critical factor.

### CHEMICAL TRANSFORMATION OF POLYOLEFINS

While incorporating weak bonds into the PE backbone confers degradability and recyclability, it inevitably compromises the inherent stability. This instability can lead to undesired degradation during the service life, potentially diminishing material performance. For instance, materials containing in-chain ester groups are susceptible to pH [104], while those with backbone carbonyls are sensitive to light [17]. Consequently, resolving the conflict between service performance and degradability poses a significant challenge. Recently, strategies focusing on after-use transformation to endow persistent PE degradability through polymer-backbone modification have gained attention [105]. These approaches maintain the excellent weatherability of conventional polyolefins during the use stage, while enabling the transformation of persistent polyolefin into main-chain-degradable polymers containing weak bonds after use.

### Degradable polyolefins from chemical transformation of PE

**Figure 5.**
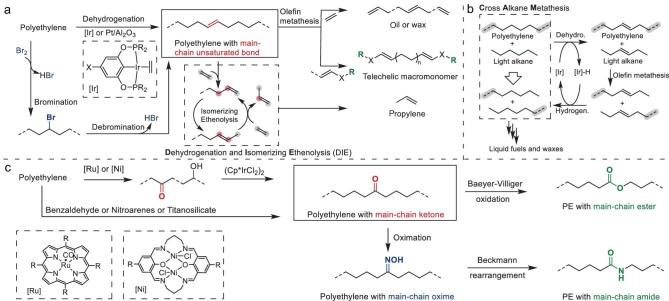
Overview of polyolefin chemical transformation. (a) Chemical recycling of PE via dehydrogenation and unsaturation-driven degradation. (b) Mechanism of cross alkane metathesis (CAM) for PE degradation. (c) Oxidation of PE producing keto-PE and upcycling via backbone rearrangement.

Given the vast global inventory and continuous production of commercial polyolefin materials, research into their intrinsic degradation pathways is highly significant, with several recent reviews systematically summarizing chemical degradation strategies for polyolefins, especially for PE [106–113]. One promising approach involves bromination followed by the dehydrobromination of waste PE or, alternatively, direct dehydrogenation, to introduce double bonds into the polymer backbone [114,115] (Fig. 5a). These unsaturated bonds then serve as reactive sites to enable advanced degradation cascades, facilitating the controlled breakdown or functionalization of the polyolefin chain (discussed in the ‘Chemical transformation of degradable polyolefins’ section).

Alternatively, catalytic oxidation can convert PE into keto-functionalized PE (keto-PE) [116–122] (Fig. 5c). However, functional group selectivity remains a major challenge for the post-polymerization modification of PE, often yielding mixtures of ketones and hydroxyl groups. The synthesis of exclusively keto-containing polymers via this route was achieved by incorporating an additional oxidation step using Cp*Ir-catalysed transfer dehydrogenation, which preserved the polymer molecular weight [123]. Alternatively, while the highly selective oxidation of PE to keto-PE can be accomplished by using benzaldehyde, it results in significant molecular-weight reduction [116]. Employing nitroarenes as substrates, the photooxidation of LDPE at 90°C produced PE with an in-chain ketone content of ≤1.2 mol%, accompanied by a slight decrease in molecular weight (*M*_n_ reduced from 15.7 to 13.3 kDa) [124]. Recently, Vos *et al.* developed a titanosilicate-catalysed C–H oxidation method that produces keto-PE with high selectivity (conversion of ≤3.4%) under mild conditions (≤100°C) [117]. Crucially, the molecular weight of the starting PE (*M*_w_ = 4 kDa) was fully retained throughout all modifications, preserving the beneficial physical properties of the base material.

Compared with fully inert PE, those bearing functional groups in the backbone exhibit enhanced degradability. The degradation of ethylene–acrylic acid (EAA) copolymer into low-molecular-weight waxes (*M*_n_ ≈ 8.3–0.3 kDa) was achieved through cerium (Ce) catalysis under visible-light irradiation (430 nm) [125]. Remarkably, this reaction proceeds in the crystalline solid state without solvent swelling (acetonitrile or water) at mild temperatures (60°C–80°C) in air. This reaction employs oxygen in air as the reactant to facilitate the near-quantitative carbon-atom recovery of EAA. Mechanistically, backbone carboxylic acid groups undergo Ce-catalysed radical decarboxylation, generating alkyl radicals along the chain that induce multiple C–C bond cleavages per –COOH group. This radical-driven strategy extends to nonfunctionalized PE [126]. More importantly, when blended with stearic acid, the solid-state photo-oxidative degradation of commercial HDPE (*M*_w_ ≈ 59.4–4.3 kDa) could be achieved under identical Ce/visible-light conditions. Here, stearic acid acts as an external radical source, in which Ce/light-generated radicals initiate hydrogen atom transfer from the PE backbone, propagating radical sites that enable high-efficiency chain scission. Crucially, both systems [125,126] demonstrate mechanochemical enhancement via ball milling, improving the degradation efficiency.

These strategies not only enhance the chemical recyclability of polyolefin waste, but also open pathways for value-added repurposing, potentially transforming inert plastic waste into degradable or chemically recyclable materials. However, compared with direct synthesis, these post-transformation methods still require improvements in conversion and selectivity to enhance the functionality. Additionally, the suppression of side reactions such as degradation remains a critical challenge.

### Chemical transformation of degradable polyolefins

PE with unsaturation or in-chain ketones serves as a platform for further transformations enabling chemical recycling (Fig. 5). Unsaturated PE could undergo primary conversion through metathesis reactions with olefin-containing substrates by using conventional Grubbs catalysts. This includes ethenolysis with ethylene to yield value-added α,ω-divinyl-functionalized oligomers [77,81,85] or reactions with functionalized olefin monomers to produce telechelic macromonomers suitable for further upcycling applications [73,75,76,114]. Additionally, the direct oxidation of unsaturated PE can generate similar telechelic macromonomers [115]. These telechelic macromonomers with hydroxyl or carboxyl/ester end groups can be repolymerized via condensation reactions to form closed-loop recyclable PE-like materials containing backbone ester linkages [49]. Furthermore, catalytic cascades combining dehydrogenation and metathesis have been developed as key strategies. These include the dehydrogenation and isomerizing ethenolysis (DIE) process for converting waste PE into propylene [127,128], as well as tandem catalytic cross alkane metathesis (CAM) employing either homogeneous small-molecule catalysts [129] or heterogeneous catalysts [130,131] to transform waste PE into saturated hydrocarbons (C_3_–C_22_). Specifically, the DIE process degrades PE into propylene through a tandem reaction involving backbone dehydrogenation, iterative double-bond isomerization and ethenolysis (Fig. 5a). Meanwhile, CAM operates via a dehydrogenation–hydrogenation cycle, first generating unsaturated small alkanes and PE through dehydrogenation, followed by metathesis-induced chain scission of the PE and subsequent hydrogenation to yield saturated hydrocarbons (Fig. 5b).

Controlled Baeyer–Villiger oxidation using *meta*-chloroperbenzoic acid converts backbone ketones into in-chain esters in yields of ≤73% [117,132]. Alternatively, these ketones can be transformed into in-chain amides via Beckmann rearrangement [117,133–135] or Boyer–Schmidt–Aube rearrangement [136]. Furthermore, alternating polyketone polymers, such as propylene/CO copolymers, also undergo backbone rearrangement to yield corresponding poly(ketone/ester) materials [137,138]. These transformations from stable PE to aliphatic esters and amides enable degradation through solvolysis, significantly enhancing their potential for chemical recycling.

These approaches fundamentally address the high-energy-consumption issues in polyolefin pyrolysis. With further cost optimization, they hold significant potential for enabling the industrial-scale degradation of commercial waste polyolefin materials.

### Chemical recycling of polyolefins

Given the global demand for polyolefins and the limitations of physical recycling, chemical recycling has emerged as a crucial approach to achieving sustainability. While conventional catalytic degradation methods such as pyrolysis and hydrogenolysis can convert polyolefins into valuable monomers (e.g. ethylene and propylene), these processes typically generate complex mixtures of byproducts, making it difficult to directly recover the original polymer. Consequently, developing degradable polyolefins that can be completely depolymerized and repolymerized is of paramount importance. There are two main types of chemical recycling: open-loop and closed-loop. Open-loop chemical recycling converts the polyolefin materials into polymers or oligomers with different structures and properties, such as the above-mentioned unsaturated-group-based and keto-based degradable polyolefins. Closed-loop recycling, on the other hand, can reconstruct the original polymer with identical structure and properties. Currently, such closed-looped recycling systems primarily rely on reversible bonds, including Si–O bonds [97] and ester linkages, which enable efficient chemical recycling.

**Figure 6.**
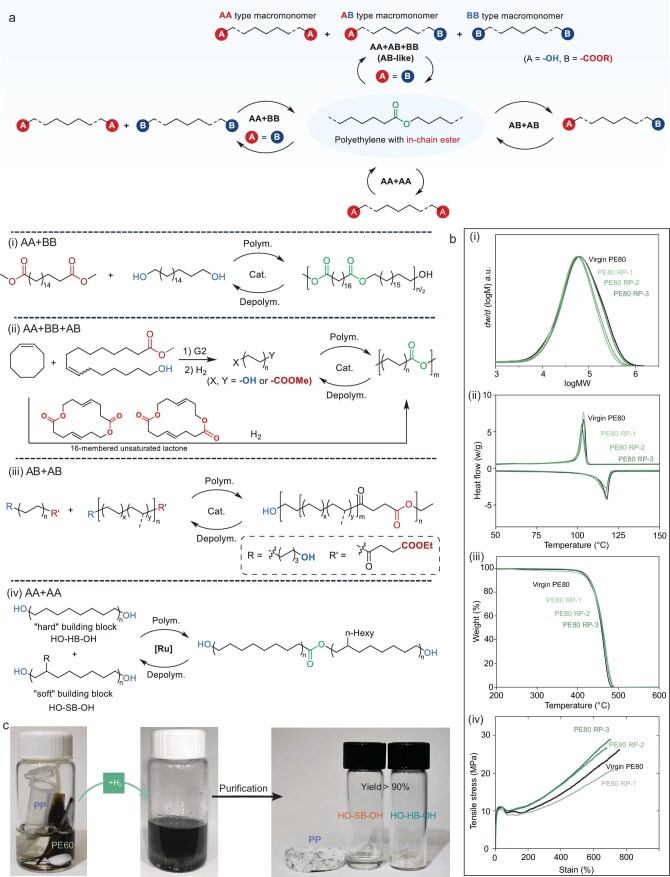
Closed-loop recyclable polyolefins based on in-chain ester. (a) Scope of four pathways to recyclable PE: (i) AA + BB, (ii) AA + AB + BB, (iii) AB + AB and (iv) AA + AA. (b) Typical properties of recycled polyolefins with in-chain esters (PE80: samples containing 80% hard block; PE80 RP-x: recycled PE80 for *x* cycles) [44], Copyright 2023, The American Association for the Advancement of Science. (i) Gel permeation chromatography (GPC) curves, (ii) differential scanning calorimeter (DSC), (iii) thermal gravimetric analyzer (TGA), (iv) tensile test. (c) Mixed-plastic recycling [44], Copyright 2023, The American Association for the Advancement of Science.

Ester-functionalized polyolefins offer four distinct recycling pathways (Fig. 6a):

- AA + BB-type macromonomer recycling systems: pioneered by Mecking and co-workers, this approach utilizes bio-based aliphatic diols or diacids/diesters monomers to construct recyclable polyolefin analogs [7,48] (Fig. 6a:i). The resulting polycarbonates and polyesters contain strategically placed ester groups along the polymer backbone that serve as cleavage sites, enabling efficient depolymerization via solvolysis with recovery yields of >96% and further transesterification polycondensation to reproduce the original polymer [7]. However, this method requires precise control of the 1 : 1 stoichiometric ratio between the functional groups during both synthesis and recycling to maintain consistent molecular weights and material properties.
- AA + AB + BB-type (AB-like) recycling systems: Hillmyer *et al.* developed a chemically recyclable system based on telechelic macromonomers with difunctional ester/hydroxyl chain terminators. This was synthesized through the ROMP of cyclooctene, employing a difunctional chain-transfer agent that contains both ester and hydroxyl groups [139] (Fig. 6a:ii). This self-balancing system maintains a constant ratio of terminal groups for transesterification polycondensation, allowing excellent retention of molecular weight in the resulting linear polymers, albeit with some compromise in elongation at break. Additionally, similar recycling systems include ester-containing polymers prepared by metathesis polymerization [65–70] and oxidation rearrangement of E/CO copolymers [117,132], which similarly yield mixtures of AA, BB and AB-type macromonomers during recycling. Among these ROMPs, by designing a cyclic comonomer with proper ring strain [70], Jian *et al.* demonstrated that ester groups could be positioned into the polymer backbone with a precise and uniform distribution, resulting in macromonomers with tailored structures and molecular weights in closed-loop recycling.
- AB-type macromonomer systems: these polymers can be cleanly depolymerized into well-defined AB macromonomers, thereby eliminating stoichiometric constraints during repolymerization. Notable examples include ester-containing polymers derived from the alkoxycarbonylation of undec-10-ene-1-ol or its derivates with CO [57,59], as well as precisely controlled AB macromonomers synthesized through CCTP and end-capping strategies [71,72].
- AA-type dehydrogenative esterification: Miyake and co-workers employed Ru [44,54] and Mn [55] catalysts to synthesize ester-functionalized polyolefins, poly-cycloolefins and block copolymers from diol monomers. Remarkably, the same catalytic systems can hydrogenate these polymers back to their diol precursors, establishing an efficient AA + AA recycling loop. This approach demonstrates exceptional property retention, with the recycled materials maintaining nearly identical molecular weights, thermal stability and mechanical properties compared with the virgin polymers (Fig. 6b).

A critical challenge in developing chemically recyclable polyolefins lies in the complex nature of real-world plastic waste, which typically consists of heterogeneous mixtures containing PE, PP, PET, nylon, etc. The selective recycling of target polymers from such mixed streams presents significant technical difficulties. The above-mentioned ester-functionalized polyolefins offer a potential solution through their selective degradability. These materials can be specifically depolymerized via the catalytic hydrolysis or solvolysis of their ester linkages, enabling efficient separation from persistent polymers such as PE, PP and polystyrene in mixed waste streams. This can be enhanced by controlling the macromonomer molecular weight or introducing branched architectures to create sufficient solubility differences for effective separation [7,44] (Fig. 6c).

However, the chemical recycling scenario becomes more complex when dealing with waste streams containing multiple cleavable polymers, such as polyesters and nylons, which also contain hydrolyzable functional groups. In these cases, the development of advanced catalytic methods with high selectivity, sequential degradation strategies or separation techniques will be essential to achieve the selective recovery of individual polymer components [54,97]. Alternatively, the development of macromonomers with tailored structures and molecular weights enabling closed-loop recycling is highly desired [70]. This remains an important area requiring focused research efforts to enable comprehensive chemical recycling solutions for real-world plastic-waste mixtures.

To date, a systematic life-cycle assessment of these novel sustainable polyolefins containing in-chain weak bonds has not been conducted. However, the industrial feasibility of solvolysis for ester-functionalized polyolefins can be reasonably supported by established PET chemical recycling methods [140,141]. Notably, these in-chain ester polyolefins exhibit significantly lower *T*_m_ (<130°C) compared with PET (∼260°C), requiring lower processing temperatures. Moreover, while PET typically requires depolymerization temperatures of >200°C, these polyolefins can be depolymerized at just 120°C–150°C, resulting in reduced energy input for both material processing and chemical recycling. Consequently, they potentially offer superior environmental friendliness and economic viability in closed-loop recycling compared with conventional PET.

## CONCLUSION AND PERSPECTIVE

Sustainable polyolefins have emerged as a pivotal research frontier in olefin polymerization, enabling the development of diverse degradable and recyclable polymers. Current synthetic strategies primarily focus on incorporating weak bonds into polymer backbones. Starting from fossil- or bio-based monomers such as ethylene, propylene, butadiene, carbon monoxide, carbon dioxide, cyclic olefins and bio-renewable diols, oleate, etc., weak bonds, including carbonyls, double bonds, esters, etc., were introduced via coordination–insertion copolymerization, metathesis copolymerization or tandem reactions involving the synthesis of telechelic macromonomers and following polycondensation. Alternatively, employing post-polymerization modifications such as oxidation, dehydrogenation, in-chain rearrangement or metathesis reactions could also impart degradability into persistent polyolefins. By incorporating in-chain cleavable bonds, these sustainable polyolefins can undergo chain scission through external stimuli such as UV light, acids, hydrolysis, solvolysis or catalysis, resulting in their degradation into oligomers or small molecules. However, only a limited number of systems, such as those containing ester linkages, currently achieve closed-loop recycling. These approaches face significant challenges, including reaction complexity, incomplete conversion, limited control over the polymer microstructure and difficulties in comonomer synthesis. Consequently, striking a balance between material costs, performance and controllable recyclability remains a critical challenge in the field. Future research efforts should prioritize the following directions:

- Highly controlled photodegradable polyolefin: PE containing sparse backbone carbonyl groups (e.g. via ethylene/CO copolymerization or post-polymerization oxidation) exhibits thermal/mechanical properties similar to those of conventional polyolefins such as HDPE. Current research predominantly focuses on synthesis, with limited studies on photodegradation. At present, PE with in-chain ketones exhibits low photodegradation efficiency, requiring prolonged irradiation (several days and even months) for significant chain scission, alongside low functional group-conversion efficiency. Future efforts should place a greater emphasis on photodegradation reactions, with the goal of augmenting their reaction efficiency and gaining better control over the structures of degradation products. Furthermore, beyond CO, which significantly poisons the coordination–insertion polymerization catalyst, there exists a pressing need to develop new comonomers that could be copolymerized with ethylene or other fundamental olefin monomers more readily. This would enable the imparting of photodegradability to polyolefins, thereby facilitating better control over polymer-chain structures. Besides, the environmental impacts of keto-based polyolefins remain poorly understood. Although these materials offer the advantage of photo-degradability, there is a striking lack of research evaluating the fate and potential reuse of their degradation byproducts, as well as their long-term ecological consequences. This gap represents a crucial research frontier that should be addressed to properly assess the sustainability claims of these materials.
- Backbone structure optimization of recyclable polyolefin: currently, the only recyclable polyolefin materials available are those incorporating heteroatoms—such as ester (C–O) or Si–O bonds—into their backbones. To address the challenges in mixed-plastics recycling, additional reversible bonds (e.g. urethane linkages) should be incorporated into polyolefin backbones to enhance the bulk polyolefin properties and optimize the degradation conditions. Furthermore, all-carbon-backbone polyolefins currently cannot undergo closed-loop recycling, but this is highly desired. Designing novel C–C-type weak-bond backbones, which are totally different from the conventional C–O, C–S, C–N and Si–O weak bonds, may resolve this challenge, achieving recyclability while preserving the intrinsic polyolefin properties. How to build all-carbon-backbone sustainable polyolefins is notably expected.
- Precise control over polymer-chain structure: achieving precise control over polymer microstructures represents a fundamental challenge in designing next-generation degradable polyolefins. Current methods for incorporating cleavable bonds along polymer chains are limited in their ability to control the bond spacing with atomic-level precision. Advanced macromonomer strategies and polymerization techniques could enable the synthesis of polyolefins with programmable architectures, facilitating systematic studies of the structure–property relationships between topology, reversible bond distribution and degradation behavior. AB-type macromonomers, namely telechelic polyolefins, are expected with the concurrent precision on molecular weight and molecular-weight distribution. This will enable the tailored synthesis of macromonomers, which are applied into macromonomer–polyolefin–macromonomer closed-loop recycling (relative to the monomer–polyolefin–monomer process).
- Development of cost-effective, high-active catalysts: catalytic systems play a pivotal role in synthesis, transformation and degradation processes. However, many of those discussed here are hampered by high costs or insufficient efficiency to be realistically considered for industrial use. Cases in point are late-transition-metal catalysts with inadequate activity for functionalized or sustainable polyolefin synthesis or the use of expensive Ir/Ru-based catalysts for PE dehydrogenation to make telechelic oligomers or propylene. Addressing these challenges requires the development of cost-effective catalytic alternatives that demonstrate superior activity, precise reaction control and scalability. These advancements are essential for transitioning polyolefin upcycling from laboratory-scale demonstrations to commercially viable processes.

The development of sustainable polyolefins will require multidisciplinary innovations that bridge catalysis, materials science and process engineering. By prioritizing controllability, scalability and performance retention, researchers can overcome the existing trade-offs and advance these materials toward practical applications in a circular economy. The field stands at a critical juncture, where fundamental advances in polymer chemistry could redefine the sustainability of plastic materials, making it an exceptionally promising area for future circular economy.
